# Combined LRRK2 mutation, aging and chronic low dose oral rotenone as a model of Parkinson’s disease

**DOI:** 10.1038/srep40887

**Published:** 2017-01-18

**Authors:** Hui-Fang Liu, Philip Wing-Lok Ho, Gideon Chi-Ting Leung, Colin Siu-Chi Lam, Shirley Yin-Yu Pang, Lingfei Li, Michelle Hiu-Wai Kung, David Boyer Ramsden, Shu-Leong Ho

**Affiliations:** 1 Division of Neurology, Department of Medicine, University of Hong Kong, Hong Kong; 2 Institute of Metabolism and Systems Research, University of Birmingham, United Kingdom

## Abstract

Aging, genetics and environmental toxicity are important etiological factors in Parkinson’s disease (PD). However, its pathogenesis remains unclear. A major obstacle is the lack of an appropriate experimental model which incorporates genetic susceptibility, aging and prolonged environmental toxicity. Here, we explored the interplay amongst these factors using mutant LRRK2^R1441G^ (leucine-rich-repeat-kinase-2) knockin mice. We found that mutant primary cortical and mesencephalic dopaminergic neurons were more susceptible to rotenone-induced ATP deficiency and cell death. Compared with wild-type controls, striatal synaptosomes isolated from young mutant mice exhibited significantly lower dopamine uptake after rotenone toxicity, due to reduced striatal synaptosomal mitochondria and synaptic vesicular proton pump protein (V-ATPase H) levels. Mutant mice developed greater locomotor deficits in open-field tests than wild-type mice following low oral rotenone doses given twice weekly over 50 weeks (half their lifespan). The increased locomotor deficit was associated with specific reduction in striatal mitochondrial Complex-I (NDUFS4) in rotenone-treated mutant but not in similarly treated wild-type mice. Our unique experimental model which incorporates genetic effect, natural aging and prolonged oral environmental toxicity administered to mutant knockin LRRK2 mice over half their life span, with observable and measurable phenotype, is invaluable in further studies of the pathogenic process and therapeutics of PD.

Parkinson’s disease (PD) is a common neurodegenerative disease characterized by loss of dopaminergic neurons in substantia nigra par compacta (SNpc) and dopamine (DA) depletion in the striatum. Current evidence indicates that its pathogenesis involves a complex interaction of aging, genetic susceptibility, and environmental factors, especially in sporadic PD^1^. Its incidence and prevalence are rare before the age of 50 years and increase substantially with aging^2^. At least 12 genes have been linked to familial PD^3^. Furthermore, whole genome studies showed genetic risks in sporadic PD cases^4^. Among these genetic risks, those of LRRK2 mutations are the most common, being found in up to 2% of sporadic cases^5^,^6^. The LRRK2^R1441G^ mutation, which is at the same location as the LRRK2^R1441C^ mutation, is located in the ROC (Ras of complex proteins) domain of the LRRK2 gene^7^,^8^,^9^,^10^, and is the most common cause of familial PD in the Basque region of Spain^11^. LRRK2-associated PD demonstrates indistinguishable clinical features from typical idiopathic cases, suggesting that they may share similar pathogenic mechanisms^12^. Hence, LRRK2 mutations are appropriate genetic models in PD.

Various therapeutic strategies have failed to modify the disease processes in PD^13^. One important reason is the lack of an appropriate experimental model which reflects the pathogenic processes in human sporadic PD^14^,^15^,^16^. Epidemiological studies have strongly suggested that environmental risk factors are involved^1^,^17^,^18^. Plantation and farm workers exposed to chronic pesticide use were found to be more likely to develop PD^19^,^20^. Indeed, a naturally occurring pesticide, rotenone, is used as a specific mitochondrial Complex-I inhibitor in experimental models of PD^16^,^21^,^22^. However, most existing toxin-based models utilize high doses of toxins administered over a short period^14^,^15^,^23^,^24^, which does not occur in most cases of PD in human. We hypothesize that the majority of cases of sporadic PD is a result of a combination of genetic susceptibility, environmental toxin exposure and aging in various proportions. This combination of factors has not been explored previously in an animal model. Hence, developing an appropriate experimental animal model involving these etiological factors is crucial to explore the early pathological processes with clinical relevance and future development of disease-modifying therapies^25^,^26^. Thus, the aim of this study is to characterize the abnormalities in a knockin (KI) mutant mouse model that incorporates these three most important risk factors: genetic susceptibility (LRRK2 KI mutation), environmental toxin (rotenone), and aging. We studied the effects of the KI mutation and toxin exposure in *in vitro* and *ex vivo* samples from prenatal and young animals, respectively. Age was then introduced as a third factor in the *in vivo* experiments.

## Results

### Mutant knockin cortical neurons were much more susceptible to rotenone-induced cell death and ATP depletion

**Figure 1:**
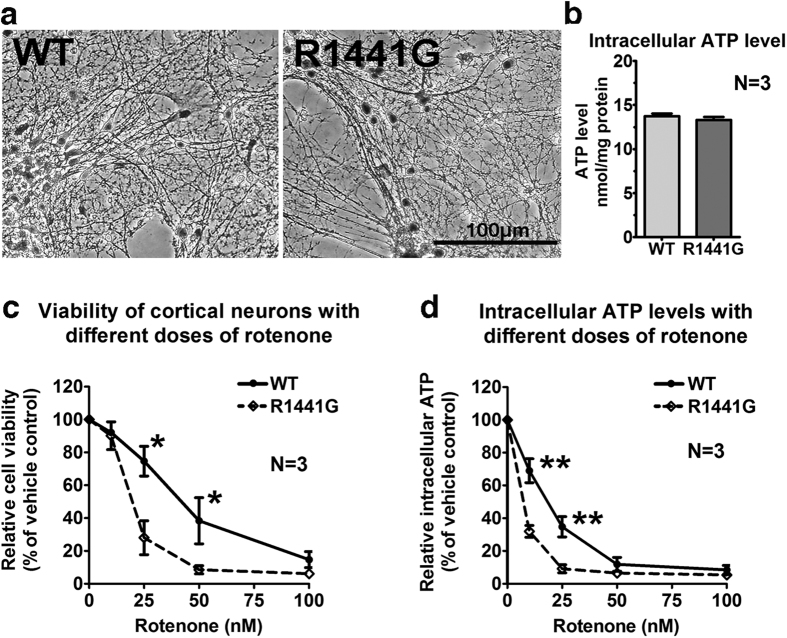
Cell viability and ATP levels of LRRK2^R1441G^ mutant mouse primary cortical neurons after rotenone exposure. Primary cortical cultures were prepared from E14.5 mutant LRRK2^R1441G^ and WT mice, and treated with 10, 25, 50, 100 nM rotenone, or vehicle (DMSO; 0.01% in culture) for 6 hr. **(a)** No morphological differences were observed between WT and mutant cortical neurons (DIV7) under normal untreated condition. Scale = 100 μm. **(b)** There were no significant differences in basal cellular ATP levels between untreated WT (*solid lines*) and mutant (*dotted lines*) cortical neurons. **(c)** Mutant cortical neurons were more susceptible to rotenone in a dose-dependent manner in MTT assay, and **(d)** ATP depletion, as compared to rotenone-treated WT group. Results are expressed as percentages relative to its corresponding vehicle-treated controls. Data represents mean ± standard error of mean (SEM) from three independent experiments (N = 3). Statistical significance between groups was analyzed by unpaired t-test. *P < 0.05; **P < 0.01.

Primary cortical neuronal cultures from LRRK2^R1441G^ KI mice had significantly lower cell viability as quantified by MTT assay and cellular ATP levels compared with wild-type (WT) after rotenone exposure for 6 hr (Fig. 1c and d). Significant differences between WT and mutant cortical neurons were observed in cell viability at 25 and 50 nM rotenone (N = 3; p < 0.05), and in ATP levels at 10 and 25 nM (N = 3; p < 0.01) rotenone. There were no significant differences in cell viability and cellular ATP levels between untreated WT and mutant cortical neurons (Fig. 1b).

### Mutant knockin mesencephalic dopaminergic (DA) neurons were more susceptible to rotenone-induced toxicity

**Figure 2:**
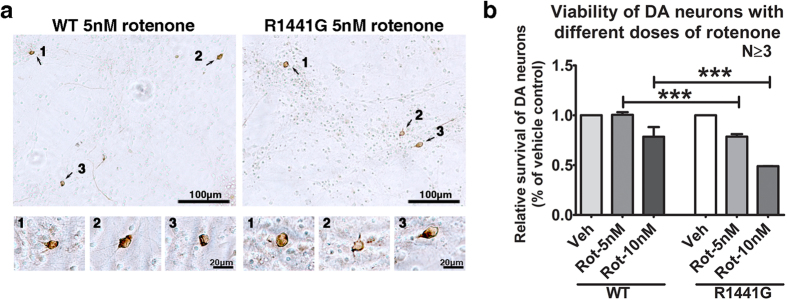
Cell viability of LRRK2^R1441G^ mutant mouse primary dopaminergic (DA) neurons after rotenone exposure. Primary mesencephalic DA neuronal cultures (DIV7) from mutant and WT mouse embryos (E14.5) were treated with 5 or 10 nM rotenone (Rot), or vehicle (Veh: DMSO; 0.01% in culture) for 48 hr. **(a)** Immunostaining of DA neurons using anti-tyrosine hydroxylase (TH) antibody after treated with 5 nM rotenone. Total number of TH-positive cells in culture was counted by two blinded observers independently under light microscope. **(b)** Relative survival of mutant DA neurons was significantly lower after exposure to rotenone than similarly treated WT groups. Data represents mean ± standard error of mean (SEM) from three independent experiments (N ≥ 3). Statistical significance between groups was analyzed by one-way ANOVA and Tukey post-hoc test, ***P < 0.001.

Mesencephalic DA neurons from E14.5 mouse embryos were treated with vehicle or rotenone (5 and 10 nM) for 48 hr. The whole culture was PFA-fixed and stained with antibody against tyrosine hydroxylase (TH; DA neuron marker). Total DA cell survival before and after treatment were assessed by manual counting of total TH^+^ cell in the whole culture by two blinded independent assessors under light microscopy (Fig. 2a). WT primary DA neurons showed similar levels of cell survival at 5 nM, and 78.5 ± 9.7% at 10 nM rotenone compared with its corresponding untreated WT control group after 48 hr (Fig. 2b). In contrast, the relative survival of mutant DA neurons had reduced to 78.5 ± 2.5% at 5 nM and 49.0 ± 0.07% at 10 nM rotenone compared to its untreated mutant control. There were significant differences in relative cell survival between WT and mutant DA neurons in both the 5 nM and 10 nM rotenone treatment groups (N ≧ 3; p < 0.001). The lower survival of mutant DA neurons following rotenone exposure is compatible with the reduced cell viability and greater ATP depletion observed in mutant cortical neurons following rotenone exposure, compared to WT.

### Increased vulnerability to rotenone in mutant striatal synaptosomes was associated with lower DA uptake, and reduced mitochondria and vesicular V-ATPase H levels in nerve terminals

**Figure 3:**
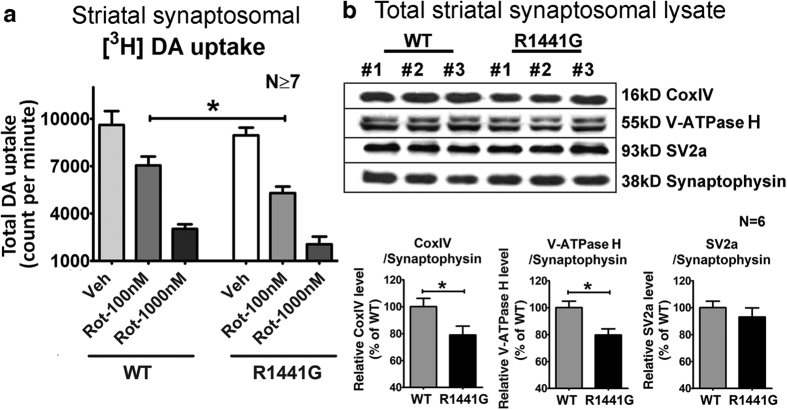
Effects of rotenone on synaptosomal [^3^H]-DA uptake, and changes in protein levels of CoxIV, V-ATPase H and SV2a in 3-month-old mouse striatal synaptosomes. (**a**) Greater reduction in LRRK2^R1441G^ mutant synaptosomal [^3^H]-DA uptake at 100 nM rotenone as compared to similarly treated WT (*WT: 7045* ± *583 cpm; mutant: 5311* ± *405* *cpm*). Data represents mean ± standard error of mean (SEM) from at least seven independent experiments. **(b)** Protein expression levels of CoxIV, V-ATPase H and SV2a in 3-month-old mouse striatal synaptosomes. CoxIV and V-ATPase H levels in total striatal synaptosomal lysates were significantly lower in mutant mice compared to WT controls (N = 6). Statistical significance between groups was analyzed using Student’s unpaired t-test, *p < 0.05.

DA uptake in striatal presynaptic terminals is known to be reduced in PD^27^,^28^. Therefore, we investigated the effects of rotenone on synaptosomal radiolabeled DA ([^3^H]-DA) uptake. Synaptosomes purified from striatum of WT and mutant 3-month old mice were exposed to 100 nM and 1000 nM rotenone and radiolabeled [^3^H]-DA uptake into the synaptosomes was quantified. Following rotenone exposure, [^3^H]-DA uptake into striatal synaptosomes was significantly lower in isolates from mutant mice compared with the results using WT isolate at 100 nM rotenone (*WT: 7045* ± *583 cpm; mutant: 5311* ± *405* *cpm; N* = *11; P* < *0.05*) but the difference did not reach significance at 1000 nM rotenone (*WT: 3034* ± *290 cpm; mutant: 2048* ± *500* *cpm; N* = *7*) (Fig. 3a). The lack of significant difference at 1000 nM rotenone may be due to excessive toxicity, as reflected by the great reduction in [^3^H]-DA uptake even in WT.

To understand why mutant striatal synaptosomes exhibited lower DA uptake than WT under rotenone toxicity, we examined for differences in expression of CoxIV (marker of the amount of mitochondria.), V-ATPase H (synaptic vesicle proton pump protein), and SV2a (a synaptic vesicle marker protein), which were normalized by synaptophysin levels (a presynaptic nerve terminal marker protein). Immunoblotting results showed similar levels of SV2a indicating similar amounts of vesicles in striatal synaptosomes isolated from WT and mutant mice. However, CoxIV and V-ATPase H expression levels were significantly lower in striatal synaptosomes from mutant mice indicating lower amounts of mitochondria and synaptic vesicle proton pump protein respectively in mutant presynaptic nerve terminals compared with WT (Fig. 3b). These results indicate that the susceptibility to rotenone, as reflected by reduced DA uptake, is due to the effects of reduced synaptosomal mitochondria and vesicle proton pump protein levels in striatal presynaptic nerve terminals of the mutant mice.

### Rotenone-treated mutant LRRK2^R1441G^ knockin mice had greater locomotor deficits compared with similarly treated wild-type mice as they aged

**Figure 4:**
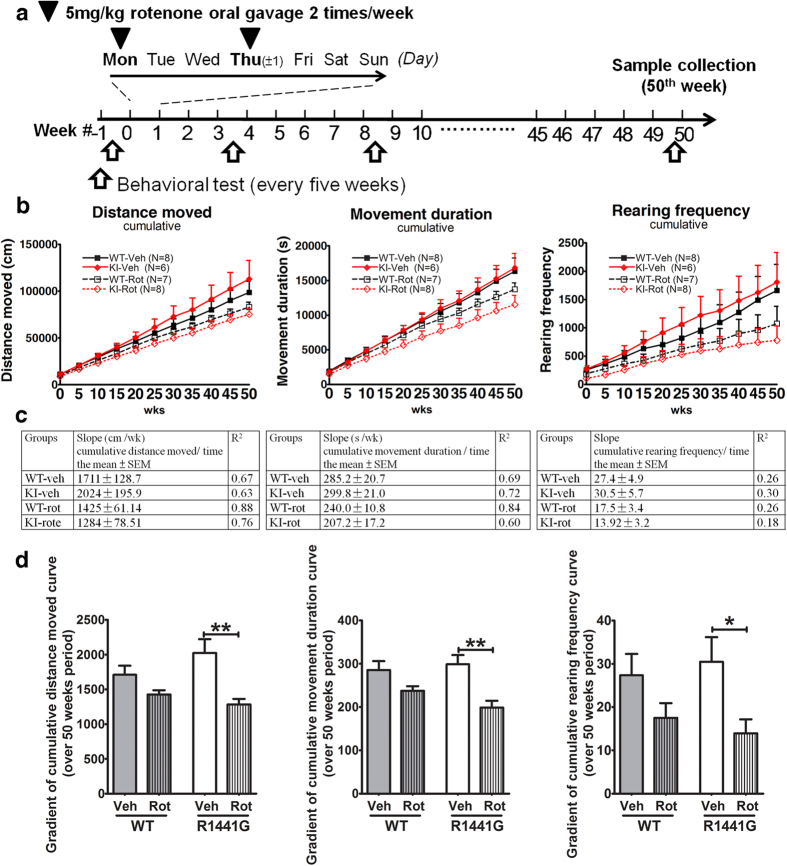
Greater reduction in locomotor activity in LRRK2^R1441G^ mutant mice after 50 weeks of low dose oral administration of rotenone (5 mg/kg). **(a)** Mice were treated orally with 5 mg/kg rotenone or vehicle (4% carboxymethylcellulose) twice per week for 50 weeks, and their locomotor activity was assessed in every five weeks. **(b)** Open-field test locomotor parameters were presented in cumulative parameter-time graphs. Each data point is expressed as mean ± standard error of mean (SEM). Three-way ANOVA showed that the combination of LRRK2^R1441G^ mutation and rotenone treatment had a significant effect on distance moved (p < 0.01), movement duration (p < 0.01) and rearing frequency (p < 0.05), with rotenone-treated mutant mice having the lowest values on all three parameters. There was a significantly greater decrease in these parameters in mutant mice treated with rotenone compared with similarly treated WT mice (Supplementary Table S1–3). **(c,d)** Gradients of cumulative distance moved, movement duration and rearing frequency of each treatment group over 50 weeks of rotenone administration. Statistical significance between groups was analyzed using Student’s unpaired t-test, *p < 0.05, **P < 0.01. (Vehicle-treated WT control group, N = 8; Vehicle-treated mutant group, N = 6; Rotenone-treated WT group, N = 7; Rotenone-treated mutant group, N = 8).

We then explored the combined effects of aging, LRRK2 knockin mutation and chronic rotenone toxicity in four sets of animals – WT vehicle-treated, WT rotenone-treated; mutant vehicle -treated; mutant rotenone-treated. Aging is inherent in this treatment regime because it spans approximately half the normal lifespan of the mice. Rotenone (5 mg/kg) was administered to 30-week old mice via bolus oral gavage twice a week for a further 50 weeks (Fig. 4a). To avoid variations in the amount of toxin exposure between each dose and individual mice, we ensured that each rotenone treatment administered via oral gavage was identical. Apart from the genotype and rotenone treatment, the other factors such as food, drink and environment were kept the same amongst all treatment groups of mice. The mice were assessed for locomotor activity (distance moved, movement duration and rearing frequency) every 5 weeks over 11 time points using open-field tests, which were recorded at similar times for 1 hr.

We analyzed the combined effects of LRRK2 mutation, rotenone treatment and aging on the mice using the cumulative locomotor parameters over 50 weeks by three-way ANOVA (Supplementary Tables S4–6). The combination of genotype with aging had no significant effect on all three locomotor parameters. However, the combination of genotype and rotenone treatment had a significant effect on distance moved (df = 1, F = 7.856, p < 0.01), movement duration (df = 1, F = 6.871, p < 0.01) and rearing frequency (df = 1, F = 3.78, p < 0.05), with rotenone-treated mutant mice having the lowest values on all three parameters. Interestingly, mutant mice treated with vehicle appeared more active than similarly treated WT mice with distance moved and rearing frequency although the differences were not significant (Fig. 4b and d). Not surprisingly, both WT and mutant mice treated with rotenone had greater deficits in all three parameters compared to those treated with vehicle. However, there was a significantly greater decrease in these parameters in mutant mice treated with rotenone compared with similarly treated WT mice (distance moved - WT: ↓ 16.7%, mutant: ↓ 36.6%; movement duration - WT: ↓ 16.8%, mutant: ↓ 35.5%; rearing frequency - WT: ↓ 36.0%, mutant: ↓ 54.3%). The parameters shown in Fig. 4b were tested against a linear fit (y = αx + β, where y = cumulative parameter and x = time (Fig. 4c). Unlike the WT mice which had similar gradients between rotenone- and vehicle-treatment, the gradients for all 3 locomotor parameters over 50 weeks for rotenone-treated mutant mice were significantly less than vehicle-treated mutant mice (Fig. 4c and d). These findings indicate that the LRRK2 mutation conferred a significant vulnerability to chronic rotenone toxicity in inducing locomotor deficits as the mice aged. Similar analyses on these locomotor parameters based on individual data point over 50 weeks of rotenone gave similar statistical results (Supplementary Fig. 1; Table S1–3)

### Rotenone-treated mutant mice had similar levels of tyrosine hydroxylase (TH) expression and apoptosis compared with similarly treated WT mice

**Figure 5:**
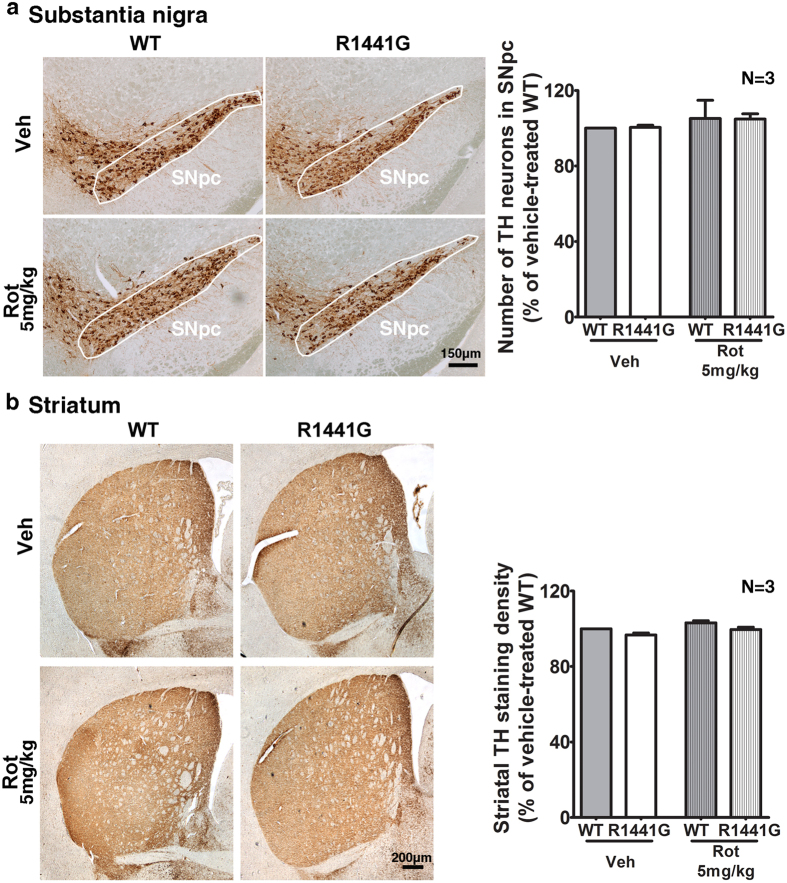
Immunohistochemistry of tyrosine hydroxylase (TH) in the substantia nigra pars compacta (SNpc) and striatum from WT and LRRK2^R1441G^ mutant mice after 50-week oral rotenone administration. Scale = 150 μm **(a)** and 200 μm **(b)**. There was no significant difference in TH-positive cell numbers in SNpc and striatal TH staining density between WT and mutant mice rotenone treatment (Student’s unpaired t-test). Data represents mean ± standard error of mean (SEM) from three independent experiments (N = 3).

**Figure 6:**
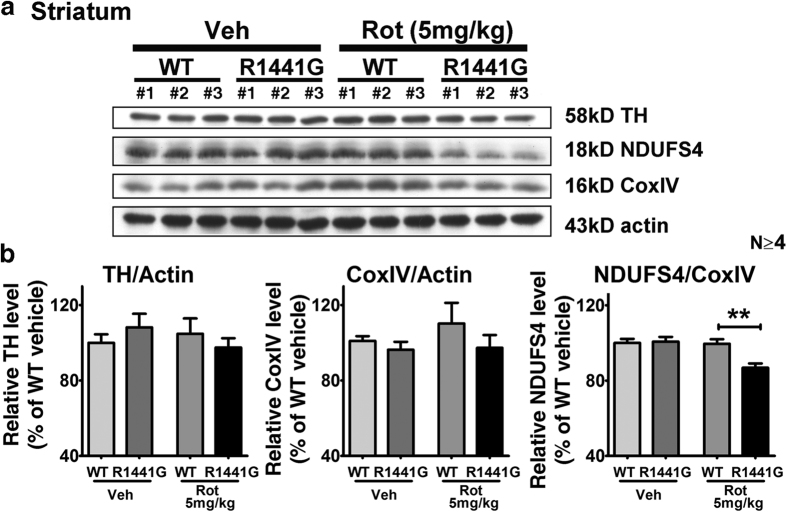
Protein expression levels of mitochondrial respiratory Complex-I subunit (NDUFS4), TH and CoxIV in mouse striatum after 50-week oral rotenone administration. Mitochondrial NDUFS4 levels were specifically decreased in mutant mice after rotenone treatment, which was not seen in those similarly treated WT mice. There were no significant changes in both TH and CoxIV levels between WT and mutant mice after rotenone treatment. Data represents mean ± standard error of mean (SEM) from at least four mice per group; Statistical significance between groups was analyzed using Student’s unpaired t-test, **P < 0.01.

Dopaminergic neurons and neurites on the substantia nigra pars compacta (SNpc) and striatum from vehicle- and rotenone-treated mice were examined following chronic rotenone treatment. Immunohistochemistry studies revealed similar TH expression in SNpc and striatum in all four groups of mice (Fig. 5). There were no observable differences in the gross structure of the dopaminergic neurons and neurites from WT compared with mutant mice, irrespective of whether the mice were treated with rotenone or not. Furthermore, similar levels of TH expression were found in total striatal lysate from mutant and WT mice, with or without rotenone treatment (Fig. 6).

**Figure 7:**
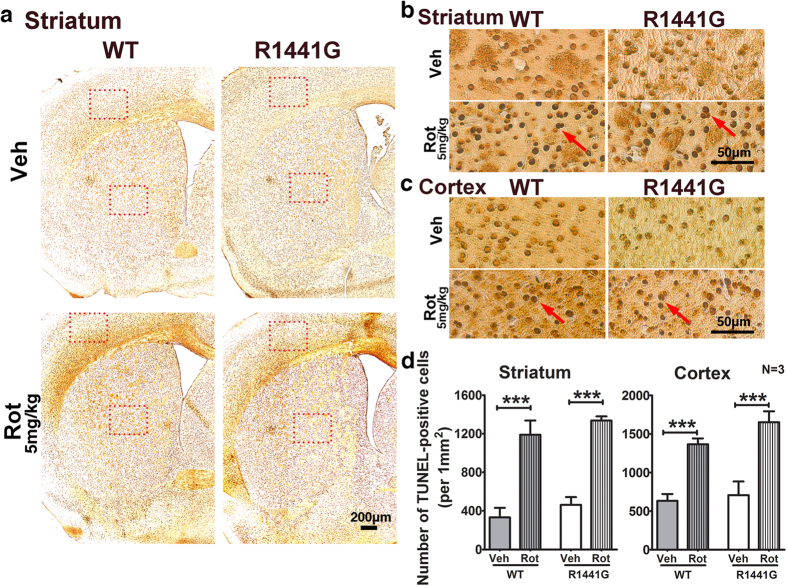
Number of apoptotic nuclei in striatum and cortex of WT and LRRK2^R1441G^ mutant mice after 50-week oral rotenone administration. (**a**) Low magnification (X40) and **(b,c)** high magnification (X400) photomicrographs in striatal and cortical regions after TUNEL assay. TUNEL-positive nuclei (*red arrow*) were seen in striatum and cortex of both WT and mutant rotenone-treated mice. N = 3. Scale = 200 μm **(a)** and 50 μm **(b,c)**. **(d)** Quantification of TUNEL-positive cells in striatum and cortex (*rectangular box*). Statistical analysis of TUNEL-positive cell numbers in stained striatum and cortex regions showed that rotenone induced similar levels of apoptosis in both WT and mutant mice. Data represents mean ± standard error of mean (SEM) from three mice per group; Statistical significance between groups was analyzed using Student’s unpaired t-test, ***P < 0.001.

**Figure 8:**
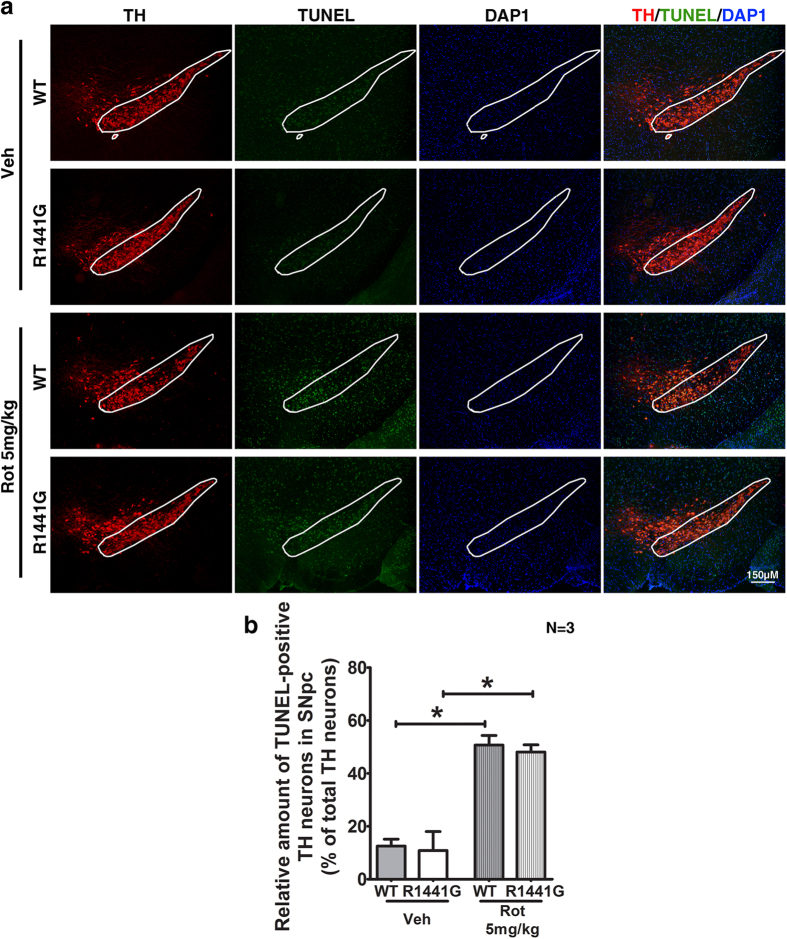
Number of apoptotic nuclei in SNpc of WT and LRRK2^R1441G^ mutant mice after 50-week oral rotenone administration. TUNEL-positive (*green*) TH-positive (*red*) dopaminergic neurons were counted in SNpc region of mice after rotenone treatment. **(a)** 50-week oral rotenone administration caused significantly greater number of TUNEL-positive DA neurons in both WT and mutant mice, as compared to their corresponding vehicle-treated groups. Scale = 150 μm. (**b**) Quantitative results of TUNEL-positive DA neurons in SNpc. Statistical analysis showed that rotenone induced similar levels of apoptosis in both WT and mutant mice. Data represents mean ± standard error of mean (SEM) from three mice per group. Statistical significance between groups was analyzed using Student’s unpaired t-test, *P < 0.05.

TUNEL staining was performed in striatum, SNpc, and cortex to assess the levels of apoptosis after rotenone treatment. Compared with vehicle-treated groups, rotenone-induced apoptosis in both mutant and WT mice was shown by markedly increased TUNEL-positive cells in these three brain regions (Figs 7a–c and 8a) as analyzed using ImageJ software (Fig. 7d). There were no significant differences in levels of apoptosis between similarly treated mutant and WT mice (Figs 7d and 8b). Although the rotenone dosage was low and did not cause significant DA cell loss, it was sufficiently effective to induce nigro-striatal and cortical apoptosis after 50 weeks of exposure which was associated with greater functional deficit in mutant striatal synaptosomal DA uptake as described above.

### Rotenone-treated mutant mice had significantly lower mitochondrial Complex-I subunit (NDUFS4) levels in striatum than similarly treated WT mice

As rotenone is a mitochondrial toxin and a specific Complex-I inhibitor, we examined for more subtle differences in striatal mitochondria after rotenone treatment. In total striatal lysate, CoxIV expression was similar between mutant and WT mice, with or without rotenone treatment. However, there was a significant reduction in mitochondrial Complex-I subunit (NDUFS4) levels in striatum from rotenone-treated mutant mice, which was not observed in rotenone-treated WT mice (N = 4; p < 0.01) (Fig. 6). In comparison, vehicle-treated WT and mutant mice had similar striatal NDUFS4 levels. These results indicated that the vulnerability conferred by LRRK2 mutation to chronic environmental toxic exposure in the form of rotenone as the mice aged was associated with reduced striatal mitochondrial Complex-I levels.

## Discussion

Aging is well-established risk factor in PD^29^,^30^. Mitochondria dysfunction is also associated with aging with more accumulated mutations^31^,^32^,^33^. However, aging and chronic exposure to environmental toxins by themselves are not the only risk factors to developing PD. There are very few population clusters of PD except in rare familial cases^34^,^35^,^36^. There are also very few conjugal cases of PD even in long lasting marriages^35^,^36^. Furthermore, monozygotic twins are more likely to develop PD than dizygotic twins, and the inheritance effect was more evident in younger onset twin-sets^37^. Epidemiological studies also showed that inheritance confers an increased risk of developing PD, with first degree relatives of PD patients having a higher risk of PD than second or third degree relatives^38^,^39^, suggesting a role of genetic susceptibility in the development of PD. However, inheritance alone is unlikely to be a significant risk factor to sporadic PD, compatible with the results obtained from our experimental model here and the existence of non-symptomatic mutant LRRK2 human carriers even in old age^40^,^41^.

PD associated with LRRK2 mutations has mostly indistinguishable features from sporadic PD, both of which tend to occur in older patients^5^,^42^. Transgenic and knockout animal models including those with mutated LRRK2 have been reported with abnormal phenotype associated with PD^43^,^44^,^45^,^46^. However, mutant LRRK2 knockin mice may more closely mimic PD because human mutant LRRK2 carriers have similar LRRK2 expression levels as those without the mutation^47^,^48^,^49^. Although most mutant LRRK2 human carriers are heterozygous, we used the homozygous knockin mouse model to accentuate the genetic risk. In our mutant mice, the R1441G mutated Lrrk2 gene is located at the intended site in the Lrrk2 locus, thus achieving biological regulation and expression patterns and levels as in WT^50^,^51^,^52^. This is in contrast to a transgenic R1441G mouse model which expressed higher LRRK2 protein level by about five to tenfold as compared to the endogenous protein in normal WT mice^53^. Despite an earlier or more robust phenotype observed, these transgenic models have their limitations that the expression pattern may not be an accurate representation of the situation in human PD^23^,^54^. Similarly, knockout mouse is genetically modified by inactivating, deleting or replacing the target gene. Whilst the knockout model is useful to explore gene function, there are no published reports of humans without LRRK2 expression.

There are three LRRK2 mutant knockin mouse models previously reported based on the R1441C^50^, R1441G^51^ and G2019S^52^ mutations. Homozygous mutant LRRK2 mice were used to accentuate the genetic risk. Despite their homozygosity, our LRRK2 mutant mice had no significant loss of dopamine neurons and locomotor deficits even in old age. However, there are increasing reports that these mutant LRRK2 knockin mice exhibit subtle abnormalities such as abnormal mitochondrial morphology^52^, different behavioral characteristics after amphetamine exposure^50^ and increased susceptibility to synaptic dysfunction even in young 3-month-old mice^51^. These findings are compatible with similar observation in humans where non-symptomatic mutant LRRK2 carriers showed abnormal DAT-SPECT^55^,^56^ and increased SN hyperechogenicity^57^,^58^.

Mitochondrial Complex-I activity is reduced in PD brain^59^,^60^, although it is unclear whether it is a primary or secondary event. We used rotenone to mimic an environmental stress to mitochondria because it exists naturally as a common organic pesticide produced by the pachyrhizus erosus plant^61^. We used different concentrations and times of exposure to rotenone between *in vitro* primary cell culture experiments and *ex vivo* striatal synaptosomal DA uptake assays is because these experiments have different aims involving different biological samples. Different biological samples have different susceptibilities to rotenone. In Fig. 1, we used primary cortical neuronal cultures from mouse embryos whereas in Fig. 3, we used striatal synaptosomes freshly isolated from young adult mice. In Fig. 1, we tested for neuronal cell viability and ATP depletion after rotenone toxicity (i.e. 0–100 nM) over 6 hours. In Fig. 3, we tested for DA uptake into striatal synaptosomes after 5 min of rotenone exposure (i.e. 100–1000 nM). Rotenone is a specific mitochondrial Complex-I inhibitor which irreversibly blocks mitochondrial ATP synthesis^62^. Although high doses of rotenone (e.g. 30 to 100 mg/kg) given repetitively over a short period (e.g. daily for 1 to 2 months) had resulted in relatively acute development of parkinsonian motor deficits and permanent damage in nigrostriatal dopaminergic neurons in various animal models^63^,^64^, they do not reflect the situation in sporadic PD which usually takes years to progress in human. Moreover, the toxicity caused by high doses of rotenone given within a short period may mask the effects of genetic susceptibility and aging. Therefore, we chose to give much lower oral doses of rotenone (5 mg/kg) over a longer period (twice weekly over half the mouse life span), which could induce apoptotic effects in the striatum of both treated WT and mutant mice without causing significant dopaminergic cell loss in the substantia nigra.

In our *in vitro* study, our mutant LRRK2 primary cortical neurons and mesencephalic DA neurons in culture were found to be more susceptible to rotenone toxicity in a dose-dependent manner (Figs 1c and d, 2b). Abnormalities in mitochondria morphology have previously been observed in striatum and cortex of mutant LRRK2 G2019S knockin mice^52^, and LRRK2 mutations can affect mitochondrial dynamics and function^65^. Since the nigrostriatal DA neurons are particularly vulnerable to energy deficiency because of their high energy-dependent autonomous pacemaker activity and vast arborization of presynaptic nerve terminals in the nigrostriatal network^66^,^67^, our findings of reduced cell viability suggested that mitochondria in mutant LRRK2 neurons may be more vulnerable to rotenone-induced dysfunction than in WT.

Next, we explored whether such susceptibility would be evident in high energy-dependent DA nerve terminals in the striatum, thus adversely affecting DA uptake. Mitochondria in synaptic terminals have unique functional properties and are more prone to damage compared with those in the cell bodies^68^. Moreover, the efficiency of ATP production and oxidative stress levels in mitochondria are modulated by different degrees of uncoupling in response to various stresses^69^,^70^,^71^,^72^,^73^,^74^. Pre-synaptic dysfunction in striatum is one of the earliest abnormalities observed in patients at the early stages of PD^75^,^76^, and in experimental PD rodent models^77^,^78^. Previously, we had shown that DA uptake in striatal synaptosomes from both three and 18-month old mice did not have significant differences between WT and mutant LRRK2 knockin mice without administration of external stress^51^. Hence, we only used three month old mutant mouse synaptosomes to study the susceptibility of LRRK2 mutation to rotenone in synaptosomal DA uptake because the use of 18-month old mice would have introduced an additional confounding factor of aging. In our *ex vivo* experiment, we found reduced DA uptake in striatal synaptosomes from mutant compared to WT mice after treatment with rotenone (Fig. 3a). We concurrently quantified, (i) CoxIV (mitochondrial cytochrome c oxidase subunit 4) levels to compare the amounts of mitochondria localized only in striatal nerve terminals (i.e. synaptosomes) between WT and mutant mice; (ii) SV2a for the amounts of synaptic vesicles; (iii) V-ATPase H for levels of vesicular proton pump; and (iv) synaptophysin levels in the synaptosomal lysates to normalize loading. Our findings showed that for the same amounts of isolated striatal synaptosomes, there were similar amounts of synaptic vesicles in mutant and WT samples but mutant synaptosomes had reduced protein levels of both CoxIV (indicating lower amounts of mitochondria) and V-ATPase H (synaptic vesicle proton pump protein) (Fig. 3b). V-ATPase H acidifies synaptic vesicles in DA nerve terminals to maintain vesicular proton gradient essential for vesicular sequestration of DA via vesicular monoamine transporter-2 (VMAT2)^79^,^80^. This acidification process is highly energy-dependent and hence is particularly susceptible to any factor which reduces energy supply, e.g. rotenone toxicity^79^,^80^. Our findings indicate that the susceptibility to rotenone in mutant mice was likely due to reduced mitochondria and vesicle proton pump protein levels localized in their nerve terminals resulting in reduced DA uptake. Our current results are compatible with our previous findings where dopaminergic synaptic function in young mutant LRRK2 mice was more vulnerable to stress induced by reserpine (VMAT2 inhibitor)^51^.

We further explored whether this vulnerability to rotenone was also found *in vivo*. Although there are previous reports involving rotenone injection in mouse models^64^,^81^,^82^, there were no similar studies using our unique oral rotenone treatment protocol for such a prolonged period of 50 weeks. Our preliminary dose finding experiments using 1 mg/kg rotenone in young WT mice given orally up to 22 weeks did not show significant difference in the locomotor parameters between rotenone-treated and untreated mice (Supplementary Fig. 2). Hence, we used a twice weekly dosing protocol with 5 mg/kg oral doses of rotenone given over 50 weeks. Although this regime is laborious and time-consuming, it more accurately reflects long-term environmental exposure to a toxin. Furthermore, aging is inherent in our model because of the chronic nature of the toxic exposure, given over half the lifespan of the mice. Using three-way ANOVA, we analyzed using the cumulative locomotor data to determine whether each factor, namely aging, genotype and rotenone treatment, was associated with reduced locomotor activity. Interestingly, the genotype (ie, LRRK2 mutation) alone was not associated with decreased locomotor activity in any of the three locomotor parameters. We took measures to minimize the effects of possible confounding factors by recording the activity at a similar time of day in the open-field tests, and observing the same batch of mice over 50 weeks of treatment. Furthermore, we only used male mice because we and others have previously shown the effects of estrogen on PD^83^, and effects of the estrus cycle on animal behavior^84^. Nevertheless, locomotor activity in individual mice is subject to large variations as would be expected with animal behavior. Hence, we analyzed the grouped cumulative locomotor parameters at 11 time points over the 50-week treatment period. Our vehicle-treated mutant mice appeared more active compared with vehicle-treated WT mice although the difference was not significant (Fig. 4b and d). However, rotenone treatment had a significant greater adverse impact on mutant mice in all three locomotor parameters compared to WT mice, to the extent that their cumulative parameters in mutant mice fell below those of WT at all 11 time points (Fig. 4b and d).

After 50 weeks of rotenone exposure, the number of TH-positive neurons in mice treated with rotenone appeared similar to those treated with vehicle in both mutant and WT mice. This result is consistent with similar TH expression in total striatal lysates from WT and mutant mice treated with either vehicle or rotenone (Fig. 6). Although there was no significant difference in TH-positive cell numbers between WT and mutant brain SNpc region after rotenone treatment, levels of apoptosis as shown by number of TUNEL-positive nuclei in both striatum and SNpc were significantly higher following rotenone compared to vehicle treatment, in both mutant and WT mice. This indicated that our chronic low oral doses of rotenone treatment was effective in inducing apoptosis in striatum, cortex, and SNpc without causing significant DA neuronal cell loss (Figs 7 and 8). Interestingly, although mitochondrial amounts in total striatal lysate as quantified by CoxIV levels were similar between mutant and WT mice after rotenone treatment, there was a significant and specific reduction in mitochondrial respiratory Complex-I component (NDUFS4) levels in striatum of mutant mice, which was not observed in rotenone-treated WT mice (Fig. 6b). NDUFS4 is a subunit of NADH dehydrogenase in the mitochondrial Complex-I assembly, and deletion of NDUFS4 reduced Complex-I function by approximately 50% in heart mitochondria^85^ and mouse embryonic fibroblasts^86^. A mouse model with midbrain-specific knockout of NDUFS4 showed impairment in DA homeostasis, and was more vulnerable to mitochondrial Complex-I toxin, MPTP^87^. Although further investigation is required to understand the detailed molecular mechanism of why there was specific reduction of NDUFS4 level in our mutant LRRK2 mice, our current findings are compatible with the specific reduction of mitochondrial Complex-I in human PD brains, and can help to explain why our mutant LRRK2 primary neurons were more susceptible to rotenone-induced ATP deficiency and cell death.

Epidemiological studies have implicated environmental factors in the pathogenesis of sporadic PD^88^,^89^. Exposure to pesticides, such as rotenone and paraquat, can cause parkinsonism in human^18^,^90^. Acute high dose exposure to such pesticides, such as suicide attempts, has been reported to cause acute parkinsonism^91^,^92^. However, the vast majority of PD patients have no such acute exposure. Although it is difficult to demonstrate direct adverse effects of environmental toxins on humans in epidemiological studies because of various confounding factors, humans are inevitably exposed to small doses of pesticides over their lifetime^93^,^94^. It is unclear what cumulative effects these pesticides pose in causing PD, especially in a genetically susceptible human. Therefore, we administered very low oral doses of rotenone regularly over half the lifespan of our mutant mice to reflect the lifelong exposure of environmental toxins in human.

In conclusion, our study showed that mutant LRRK2^R1441G^ primary cortical and mesencephalic dopaminergic neurons were more susceptible to rotenone-induced toxicity than WT neurons. Lower DA uptake in striatal synaptosomes from mutant LRRK2 mice rotenone toxicity was associated with significantly less mitochondria and V-ATPase H protein localized in the nerve terminals compared with WT mice. The susceptibility of these mutant mice to rotenone-induced mitochondrial dysfunction was associated with more severe locomotor deficits in open-field tests. Although both mutant and WT mice had similar levels of DA cell number and apoptosis, the mutant mice had specific reduction in striatal NDUFS4 (mitochondrial Complex-I subunit) levels post-treatment, which was absent in the similarly treated WT mice. Our findings indicates that genetic susceptibility in the form of LRRK2 mutation conferred a significant vulnerability to long-term environmental toxicity as the mice aged over half their lifespan, involving reduced striatal mitochondrial Complex-I levels. Our unique experimental model of PD incorporates genetic effect, natural aging and prolonged oral environmental toxicity administered to mutant knockin LRRK2 mice over half their life span, with observable abnormalities that are compatible with those observed in human PD. This model is invaluable in further studies to explore the early pathogenic changes and disease modifying therapies of PD.

## Materials and Methods

### Animals

Homozygous mutant LRRK2 knockin mice carrying point mutation (c. 4321 C > G) of LRRK2 and their wildtype littermates were used for preparation of primary neuronal cultures and *in vivo* rotenone oral gavage experiments. Our mutant LRRK2^R1441G^ knockin mice were previous described and published^26^,^51^. Similar to other LRRK2 knockin mouse models^50^,^52^, there was no overt phenotypic difference between WT and mutant LRRK2^R1441G^ mice even when these mice reached their old age of 18 months old without any external stress^51^. Our mutant LRRK2^R1441G^ mice were back-crossed with wildtype C57BL/6 N mice for over eight generations, and maintained under such genetic background thereafter. All mice were maintained on a 12 hr light/dark cycle, with lights on at 7 AM in the Laboratory Animal Unit, University of Hong Kong, which is accredited by the Association for Assessment and Accreditation of Laboratory Animal Care International (AAALAC). All experimental protocols were performed in accordance with the National Institute of Health Guide for the Care and Use of Laboratory Animals, and were approved by the Committee on the Use of Live Animals in Teaching and Research (CULATR) of the University of Hong Kong (CULATR#3452-14).

### Rotenone oral gavage

Rotenone (Sigma #R8875) was dissolved in 4% carboxymethylcellulose (Sigma #C5678). Animals (30 week-old male mice) were given a 5 mg/kg rotenone dose or vehicle twice weekly for 50 weeks. The rotenone solution and vehicle were administered by bolus oral gavage. Animals were weighed each week. Mice were subjected to open-field test every five weeks to detect the change of locomotor activity at 11 time points over 50 weeks.

### Primary cell culture and treatments

Ventral mesencephalic and cortical tissues from 14–15 day embryos were excised, and were cut into small pieces in cold EBSS (Sigma) containing 100 units/ml of penicillin and 100 μg/ml of streptomycin. Neuronal cells were dissociated by the Papain Dissociation System (Worthington Biochemical Corporation) according to manufacturer’s protocols. After dissociation, cells from the mesencephalon and cortex were seeded on poly-L-lysine pre-coated coverslips (3 × 10^6^ cells/well) or 24-well plates (0.5 × 10^6^ cells/well) respectively.

Mesencephalic neurons were cultured in Dulbecco’s Modified Eagles Medium (DMEM) supplemented with 20% FBS at 37 °C in an atmosphere of 5% CO_2_/95% air. The medium was changed on the 2^nd^ day *in vitro*. On the 4^th^ and 6^th^ days *in vitro* half of the medium was replaced with serum-free DMEM containing 0.02 ml/ml B27 (Gibco). AraC (5 μM; Sigma) was added from 4^th^ day *in vitro*. The mesencephalic neurons was treated with rotenone (5 and 10 nM) or vehicle from 7^th^ day *in vitro* for 48 hr, then fixed with 4% paraformaldehyde (PFA) for TH immunocytochemistry staining.

Cortical cells were cultured in neural basal medium (Gibco) with 2% B27 and 5% FBS at 37 °C in an atmosphere. On 2^th^ DIV, medium was replaced with 5 μM AraC serum-free medium. Half of the medium was replaced with serum-free medium twice per week. Cortical neurons on the 8^th^ day *in vitro* were treated with either vehicle or rotenone (10, 25, 50 and 100 nM) for 6 hr, then assayed for ATP content and for cell viability or survival.

### MTT cell viability assay

Cortical neurons in 24-well plates were added 3-(4, 5-dimethylthiazolyl-2)-2, 5-diphenyltetrazolium bromide (MTT) solution to achieve final concentration of 0.5 mg/ml and a final volume of 300 μl/well. After this the plate was incubated at 37 °C for 1 hr. Formazan crystals, which developed inside each well, were dissolved by addition of DMSO (300 μl) giving a purple solution. Then the purple solution was transferred to 96-well plate 100 μl/well, and the intensity of this was determined by assaying absorbance at 570 nm using a CLARIOstar microplate reader (BMG LABTECH). Cell viability was regarded as directly related to absorbance. The experiments were repeated three times in triplicate for each experiment.

### ATP assay

ATPlite™ Luminescence Assay System (PerkinElmer) was used to measure the intracellular ATP level according to the manufacturer’s protocol. Assay samples were transferred to wells of F96 MicroWell™ White Polystyrene Plates (Nunc). The luminescence, which represents the amount of ATP present in each sample, was measured by CLARIOstar microplate reader. The experiments were repeated three times in triplicate for each experiment.

### Synaptosomal [^3^H]-dopamine uptake assay

The synaptosomal [^3^H]-dopamine uptake was assayed as previously described^51^. Briefly, synaptosomes from mice striatum were isolated. To test the effect of rotenone, synaptosomes and rotenone (100 nM, 1000 nM) or vehicle were incubated in Krebs–Ringer buffer (120 mM NaCl; 4.8 mM KCl; 1.3 mM CaCl_2_; 1.2 mM MgSO_4_; 1.2 mM KH_2_PO_4_; 25 mM NaHCO_3_; 6 mM glucose; pH 7.6) with 0.1 μM [^3^H]-dopamine for 5 min at 37 °C. Dopamine uptake was stopped by addition of cold Krebs–Ringer buffer (200 μl); the reaction solution was filter by a UniFilter^®^-96 GF/C filter, after washing and drying; [^3^H] Radioactivity (count per minute, cpm) was measured using a TopCount^®^ NXTTM microplate scintillation & luminescence counter (Packard). Non-specific uptake was determined in the presence of 10 μM nomifensine (DAT inhibitor). Specific [^3^H]-dopamine uptake was calculated as the difference between [^3^H] radioactivity in tested sample and paired negative control. Each treatment group was run in duplicate.

### Histology

Mice under anesthesia were perfused with cold PBS followed by 4% PFA. Whole brain was removed and post-fixed in 4% PFA at 4 °C overnight. After dehydration, clearing, paraffin infiltration and embedding, the midbrain (including substantia nigra) and striatum regions were sectioned coronally at 8 μm thickness.

### Immunohistochemistry

Paraffin tissue sections after antigen retrieval or cell cultures on coverslips were incubated with anti-TH antibody (1:500, Millipore #AB318) to determine the number of dopaminergic cells. Briefly, samples were incubated with primary antibody at 4 °C overnight in blocking TBS buffer containing 2% normal serum and 5% BSA. Samples were exposed to secondary antibody conjugated to HRP (Dako) for 1 hr at room temperature, and then HRP’s chromogenic substrate DAB (Dako) added. Color development was monitored and photographed under light microscope. Stained coronal sections and primary cells on coverslips were dehydrated, cleared by xylene, and mounted with Richard-Allan Scientific™ Mounting Medium (ThermoFisher). Four consecutive sections taken from three individual mice in each treatment group were used to quantify the average total number of dopaminergic neurons in SNpc region. Optical intensity after DAB staining of tyrosine hydroxylase in striatum was measured by ImageJ software (http://rsbweb.nih.gov/ij/plugins/track/track.html). Dopaminergic neurons on the coverslips were counted under blinded conditions by two independent observers. Each cell culture treatment was carried out in triplicate with a minimum of three experiments.

### Terminal deoxynucleotidyl transferase dUTP nick end labeling (TUNEL) assay

Paraffin tissue sections after antigen retrieval were assessed for the level of apoptosis using the TUNEL assay (*in situ* cell death detection kit, Roche) following the manufacturer’s protocol. Sections were incubated in 37 °C in dark for 1 hr, then washed 3 times in PBS. For DAB-stained TUNEL assay in cortex and striatum, the resultant stained slides were dehydrated, cleared by xylene, and mounted with Richard-Allan Scientific™ Mounting Medium (ThermoFisher). For immunofluorescent TUNEL assay in substantia nigra, the stained slides were incubated in 0.1% Sudan Black B before mounted with ProLong^®^ Diamond Antifade Mountant (ThermoFisher). Random images were acquired from four consecutive sections taken from three individual mice in each group. Four randomly chosen regions in each photomicrograph were used to quantify the average number of TUNEL-positive cells for striatum and cortex region, whereas TUNEL-positive dopaminergic neurons in SNpc were counted under blinded conditions by two independent observers.

### Locomotor activity test

Locomotor activity of mice was monitored using open field tests as described by^51^. The mice were kept in the experimental room for 3 days before testing. The mice moved freely in a plastic arena (26 × 26 × 40 cm), and were tracked for 1 hr using the EthoVision 3.0 system (Noldus Information Technology) at a similar time of day. Movement duration (seconds), distance moved (cm), rearing frequency (number of times standing on hind legs) of each mouse over 1 hr was recorded at 11 points over 50 weeks.

### Immunoblotting

Mouse striatum was dissected and homogenized in cold lysis buffer (Cell signaling) with protease inhibitor cocktail (Roche). Protein lysates were incubated on ice for 20 min and clarified by centrifugation at 4 °C for 15 min at 14,000 rpm. Protein concentration was determined by the Bradford assay, and stored at −80 °C. Equal amounts of protein were electrophoresed in 10%, 12 and 15% SDS-polyacrylamide gels at 100 volts for 1.5 to 2 hr and electro-transferred to nitrocellulose membranes. Membranes blot was probed with primary antibody, and followed by HRP-conjugated secondary antibodies (DAKO). Immunoblot results were visualized on X-ray film using Amersham ECL Western Blotting Detection Reagent (GE Lifesciences). Intensity of the various protein bands was quantified using ImageJ software (http://rsbweb.nih.gov/ij/plugins/track/track.html). COX IV (1:2000, Abcam #ab16056, 16 kD), NDUSF4 (1:500, Santa-Cruz #sc-100567, 18 kD), TH (1:2000, Millipore #MAB318, 58 kD), Actin (1:500, Santa-Cruz #sc-1615, 43 kD), Synaptophysin (1:2000, Cell Signaling #D35E4, 38 kD), V-ATPase (1:1000, ThermoFisher #PA5-22134, 55 kD), SV2a (1:1000, Santa-Cruz #sc-11936, 93 kD) were the primary antibodies used. Mice (4 to 6 mice) were used to detect the TH and mitochondria protein levels in each group. Six mice were used to detect the mitochondria and V-ATPase levels in striatal synaptosomes.

### Statistical analysis

Data are expressed as mean ± standard error mean (SEM). Student’s t-test (Prism software package, GraphPad Software Inc.) was used to compare levels of protein expression; one-way ANOVA (Prism software package, GraphPad Software Inc.) was used to compare DA neuron viability; Student’s t-test and three-way ANOVA in the SPSS package (IBM) was used to analyze the locomotor activity in the open-field tests. For the three-way ANOVA, the raw and cumulative data (from 11 time points over 50 weeks) from the locomotor activity parameters (distance moved, movement duration and rearing frequency) were analyzed separately. Differences between groups were considered significant at p < 0.05.

## Additional Information

**How to cite this article**: Liu, H.-F. *et al*. Combined LRRK2 mutation, aging and chronic low dose oral rotenone as a model of Parkinson’s disease. *Sci. Rep.* **7**, 40887; doi: 10.1038/srep40887 (2017).

**Publisher's note:** Springer Nature remains neutral with regard to jurisdictional claims in published maps and institutional affiliations.

## Supplementary Material

Supplementary Information
