# Supplementary material for: Combined LRRK2 mutation, aging and chronic low dose oral rotenone as a model of Parkinson’s disease
Source: Sci Rep. 2017 Jan 18;7:40887. doi: 10.1038/srep40887 (PMC5241661; doi:10.1038/srep40887)
Supplement: Supplementary Information [file srep40887-s1.pdf]

Supplementary Information:

Combined LRRK2 mutation, aging and chronic low dose oral rotenone as a model of Parkinson's disease

Hui-Fang Liu[1*], Philip Wing-Lok Ho[1*], Gideon Chi-Ting Leung[1], Colin Siu-Chi Lam[1], Shirley Yin-Yu Pang[1], Lingfei Li[1], Michelle Hiu-Wai Kung[1], David Boyer Ramsden[2], Shu-Leong Ho[1#]

1. Division of Neurology, Department of Medicine, University of Hong Kong, Hong Kong
2. Institute of Metabolism and Systems Research, University of Birmingham, United Kingdom

* These authors contributed equally in this paper

Correspondence and requests for materials should be addressed to:
Prof. Shu-Leong Ho
Division of Neurology, Department of Medicine,
University of Hong Kong, Queen Mary Hospital,
Hong Kong.
Tel.: (852) 2855 3315
Fax: (852) 2974 1171
Email: slho@hku.hk

Supplementary Figure S1

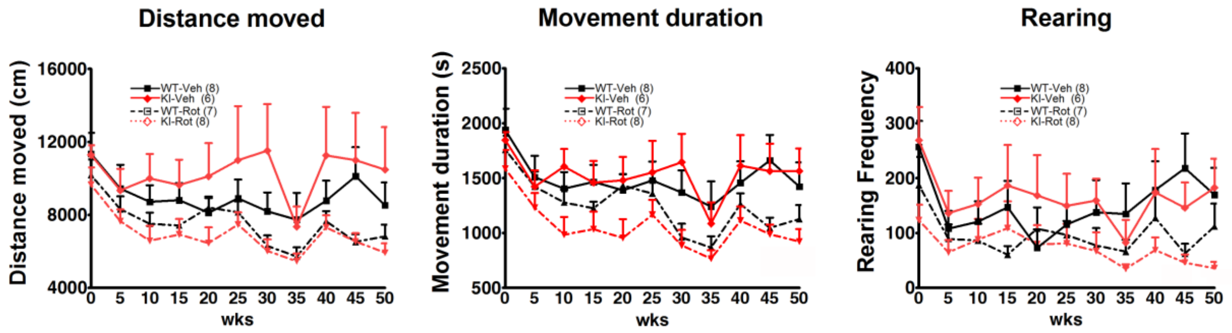

Supplementary Figure S1. Raw data of open-field locomotor activity test in four groups of mice [vehicle-treated WT (N=8); vehicle-treated KI (N=6); rotenone-treated WT (N=7); and rotenone-treated KI (N=8)] are presented in parameter-time graphs over 50 weeks at 11 time points. This data set was used to generate the cumulative parameter-time in Fig. 4b. Data are expressed as mean ± SEM.

Supplementary Figure S2

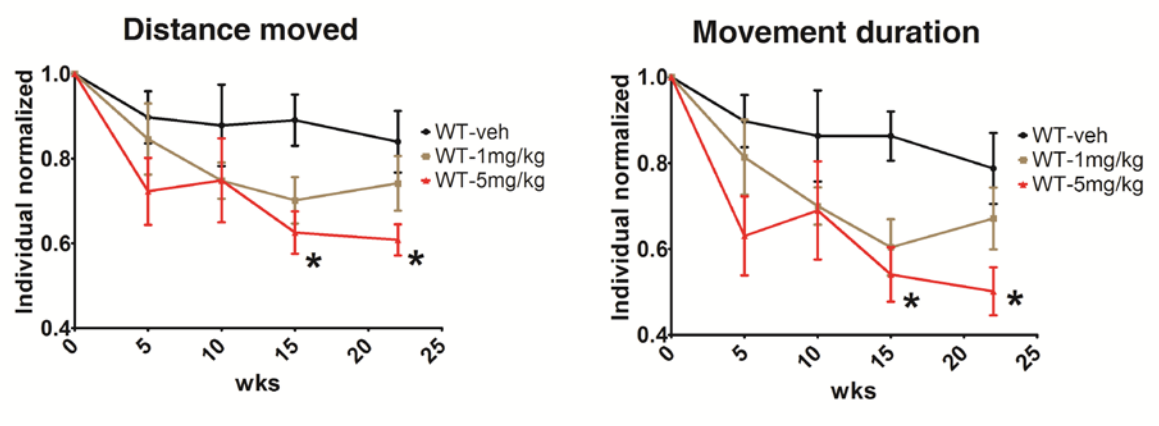

Supplementary Figure S2. Preliminary rotenone dose determination in 30-week-old wild-type C57/BL6 mice. Mice were treated orally with 1, 5 mg/kg rotenone, or vehicle twice per week for 22 weeks, and locomotor activity of each mouse was assessed by open field test every five weeks. Oral rotenone treatment at 5 mg/kg after 15 weeks caused significant reduction in both distance moved and movement duration as compared with the vehicle-treated controls ($p<0.05$). Data (mean ± SEM) was analyzed using Student’s unpaired t-test; *, $P<0.05$.

Supplementary Figure S3

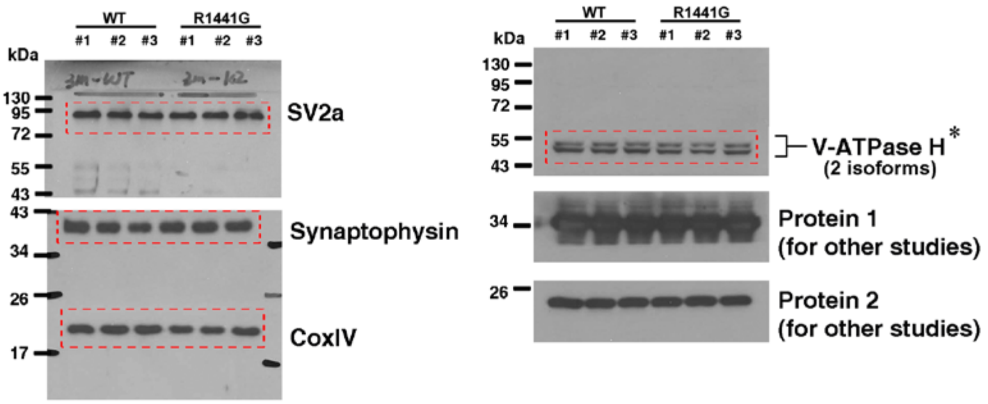

Table showing the raw band intensity measurements by Image J

		SV2a	Synaptophysin	COX IV	V-ATPase H
WT	#1	16130.55	24790.19	18205.12	18158.38
	#2	14100.09	21005.24	19890.95	16703.92
	#3	13010.24	17589.31	19348.24	16948.74
R1441G	#1	14119.07	22146.77	14478.7	14894.55
	#2	14179.24	23050.6	14130.87	12518.72
	#3	18026.33	21873.65	16258.75	15306.67

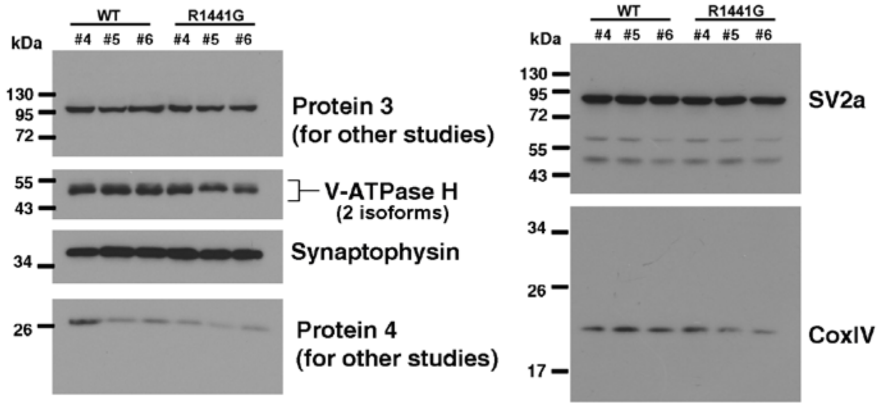

Table showing the raw band intensity measurements by Image J

		SV2a	Synaptophysin	COX IV	V-ATPase H
WT	#4	20255.45	19866.02	8406.024	17416.38
	#5	20622.16	24819.84	12086.34	19926.14
	#6	19135.62	24164.14	9226.803	18506.31
R1441G	#4	19232.92	25704.5	10561.17	16789.89
	#5	19260.62	23619.02	5524.368	13150.17
	#6	18296.14	21297.14	3925.296	12056.53

Supplementary Figure S3. The raw scans of full length western blots as shown in Figure 3.

The hatched red lines indicate the correct sized bands of the target proteins in Figure 3.

* Both isoform bands of V-ATPase H were used for quantification.

Supplementary Tables

Supplementary Table S1

Tests of Between-Subjects Effects

Dependent Variable: **Distance moved**

Source	Type III Sum of Squares	df	Mean Square	F	Sig.
Corrected Model	878981949.354[a]	43	20441440.683	2.144	.000
Intercept	22221726121.661	1	22221726121.661	2331.043	.000
Genotype	7121599.627	1	7121599.627	.747	.388
Rotenone	463784093.742	1	463784093.742	48.651	.000
age	241555789.485	10	24155578.948	2.534	.006
Genotype * Rotenone	78679492.202	1	78679492.202	8.253	.004
Genotype * age	29842175.457	10	2984217.546	.313	.977
Rotenone * age	51410814.984	10	5141081.498	.539	.862
Genotype * Rotenone * age	31145229.539	10	3114522.954	.327	.974
Error	2621562862.737	275	9532955.864		
Total	25509737123.996	319			
Corrected Total	3500544812.091	318			

a. R Squared = .251 (Adjusted R Squared = .134)

Supplementary Table S2

**Tests of Between-Subjects Effects**					
Dependent Variable: **Movement duration**					
Source	Type III Sum of Squares	df	Mean Square	F	Sig.
Corrected Model	24346509.057[a]	43	566197.885	2.810	.000
Intercept	555040341.586	1	555040341.586	2754.516	.000
Genotype	470226.158	1	470226.158	2.334	.128
Rotenone	10145727.817	1	10145727.817	50.351	.000
age	9373925.418	10	937392.542	4.652	.000
Genotype * Rotenone	1188017.307	1	1188017.307	5.896	.016
Genotype * age	521667.314	10	52166.731	.259	.989
Rotenone * age	1436934.470	10	143693.447	.713	.712
Genotype * Rotenone * age	696502.967	10	69650.297	.346	.968
Error	55413039.995	275	201501.964		
Total	633180076.400	319			
Corrected Total	79759549.052	318			
a. R Squared = .305 (Adjusted R Squared = .197)					

Supplementary Table S3

**Tests of Between-Subjects Effects**					
Dependent Variable: **Rearing frequency**					
Source	Type III Sum of Squares	df	Mean Square	F	Sig.
Corrected Model	948339.461[a]	43	22054.406	1.710	.006
Intercept	4591728.420	1	4591728.420	356.038	.000
Genotype	3492.199	1	3492.199	.271	.603
Rotenone	423717.275	1	423717.275	32.855	.000
age	284718.822	10	28471.882	2.208	.018
Genotype * Rotenone	31399.174	1	31399.174	2.435	.120
Genotype * age	70977.898	10	7097.790	.550	.853
Rotenone * age	74322.334	10	7432.233	.576	.833
Genotype * Rotenone * age	50431.237	10	5043.124	.391	.950
Error	3546602.357	275	12896.736		
Total	8984735.000	319			
Corrected Total	4494941.818	318			
a. R Squared = .211 (Adjusted R Squared = .088)					

Supplementary Table S1-3. Raw data of open-field test are analyzed by three-way ANOVA (open-field test parameters: distance moved, movement duration and rearing frequency). Aging and rotenone alone had a significant effect on distance moved, movement duration and rearing frequency. And genotype (LRRK2 mutation) alone had no significant effect on these three parameters. The combination of genotype with

aging also had no significant effect on these three locomotor parameters. However, the combination of genotype and rotenone treatment had a significant effect on distance moved and movement duration, but not rearing frequency.

Supplementary Table S4

Tests of Between-Subjects Effects

Dependent Variable: **Cumulative distance moved**

Source	Type III Sum of Squares	df	Mean Square	F	Sig.
Corrected Model	228029905135.108[a]	43	5303021049.654	16.375	.000
Intercept	842831916132.613	1	842831916132.613	2602.511	.000
age	209825572372.040	10	20982557237.204	64.790	.000
Genotype	30967575.283	1	30967575.283	.096	.757
Rotenone	12917522064.256	1	12917522064.256	39.887	.000
Age*genotype	191828436.402	10	19182843.640	.059	1.000
Genotype * Rotenone	2544340283.628	1	2544340283.628	7.856	.005
Age * Rotenone	4908539996.381	10	490853999.638	1.516	.133
Age * Genotype * Rotenone	974441765.826	10	97444176.583	.301	.981
Error	89059663939.305	275	323853323.416		
Total	1154553264755.257	319			
Corrected Total	317089569074.414	318			

a. R Squared = .719 (Adjusted R Squared = .675)

Supplementary Table S5

Tests of Between-Subjects Effects					
Dependent Variable: **Cumulative movement duration**					
Source	Type III Sum of Squares	df	Mean Square	F	Sig.
Corrected Model	5588773709.888[a]	43	129971481.625	18.298	.000
Intercept	21549149074.448	1	21549149074.448	3033.862	.000
age	5114022482.304	10	511402248.230	71.999	.000
Genotype	27711430.478	1	27711430.478	3.901	.049
Rotenone	277144696.595	1	277144696.595	39.019	.000
Age*genotype	3491723.268	10	349172.327	.049	1.000
Genotype * Rotenone	48806643.810	1	48806643.810	6.871	.009
Age * Rotenone	109101774.419	10	10910177.442	1.536	.126
Age * Genotype * Rotenone	13956560.759	10	1395656.076	.196	.996
Error	1953291091.961	275	7102876.698		
Total	29065057864.440	319			
Corrected Total	7542064801.849	318			
a. R Squared = .741 (Adjusted R Squared = .701)					

Supplementary Table S6

Tests of Between-Subjects Effects					
Dependent Variable: **Cumulative rearing frequency**					
Source	Type III Sum of Squares	df	Mean Square	F	Sig.
Corrected Model	56847664.035[a]	43	1322038.698	3.112	.000
Intercept	179567498.188	1	179567498.188	422.746	.000
age	39172501.049	10	3917250.105	9.222	.000
Genotype	3640.635	1	3640.635	.009	.926
Rotenone	13165485.958	1	13165485.958	30.995	.000
Age*genotype	184024.235	10	18402.423	.043	1.000
Genotype * Rotenone	1605495.503	1	1605495.503	3.780	.049
Age * Rotenone	3520834.550	10	352083.455	.829	.601
Age * Genotype * Rotenone	248121.840	10	24812.184	.058	1.000
Error	116810313.952	275	424764.778		
Total	348811853.000	319			
Corrected Total	173657977.987	318			
a. R Squared = .327 (Adjusted R Squared = .222)					

Supplementary Table S4-6. Cumulative data of open-field test are analyzed by three-way ANOVA. Aging and rotenone alone had a significant effect on cumulative distance moved, movement duration and rearing frequency. And the effect of genotype alone on cumulative movement duration just reached significance but not

cumulative distance moved or rearing frequency. The combination of genotype with aging also had no significant effect on these three locomotor parameters. However, the combination of genotype and rotenone treatment had a significant effect on these three cumulative locomotor parameters.
